# Factors associated with the utilization of inactivated polio vaccine among children aged 12 to 23 months in Kalungu District, Uganda

**DOI:** 10.1093/heapol/czaa099

**Published:** 2020-11-09

**Authors:** Mirembe Rachel Faith, Babirye Juliet, Nathan Tumuhamye, Tumwebaze Mathias, Emma Sacks

**Affiliations:** c1 Ministry of Health, Uganda Sanitation Fund Programme, Kampala, PO Box 7272, Uganda; c2 Makerere University, School of Public Health, Kampala, Uganda; c3 Bishop Stuart University, Mbarara, Uganda; c4 Johns Hopkins School of Public Health, Baltimore, Maryland, USA

**Keywords:** IPV, polio, vaccinations, immunization, children, Uganda

## Abstract

Uganda officially introduced the inactivated polio vaccine (IPV) in May 2016 as part of the polio eradication strategy and integrated it into its routine immunization programme in addition to the oral polio vaccine. The current coverage stands at 60% as of July 2017. We therefore aimed to determine factors associated with the uptake of IPV among children in Kalungu District so as to inform the implementation of the vaccine policy. A community-based cross-sectional study was conducted among caregivers of 406 eligible children aged 12–23 months through multi-stage systematic sampling and a standardized semi-structured questionnaire. Nine key informant interviews were conducted through purposive selection of health care providers and members of Village Health Teams (VHTs) based on their expertize. Modified Poisson regression and thematic content analysis were used to determine factors significant to IPV uptake among children. 71% of sampled children aged 12–23 months had received IPV in Kalungu District. The survey found that being encouraged by health workers and VHTs was significant to children’s uptake of IPV (Adjusted PR 1.24, 95% CI; 1.22–3.47). Distance to the immunization point (Adjusted PR 0.32,95% CI; 0.16–0.62) and caregiver’s education level (Adjusted PR 1.16,95% CI; 1.05–2.22) were also associated with IPV uptake. Qualitative findings from health workers and VHT members further confirmed the perception that distance to the immunization post was important, and VHTs also stated that being encouraged by health workers was critical to IPV uptake. The current prevalence of IPV uptake among children aged 12–23 months in Kalungu is 71%, higher than the last reported national coverage (60%), though still below the recommended national coverage of 95%. Efforts should be focused on sensitization of caregivers through health workers and VHTs. Immunization outreach should be strengthened so as to bring services closer to patients.

## Introduction

Over the last two decades, eradicating polio has been a key global health goal (Parker et al., 2015; WHO, 2016a). Between 2013 and 2014, the number of wild polio cases reported globally reduced from 256 to 171 and, in 2018, further reduced to 33 reported cases (WHO, 2018). Most of these outstanding cases are from three countries that have never stopped polio transmission (Nigeria, Afghanistan and Pakistan). Tackling the last 1% of polio cases has still proven difficult due to reasons such as conflict, political instability, hard-to-reach populations, community misconceptions and poor infrastructure (Omole et al., 2015).

**KEY MESSAGES**

- Over the last two decades, eradicating polio has been a key global health goal, and the existence of wildtype virus with use of oral polio has catalysed countries to add inactivated polio vaccine (IPV) to their schedules. Uganda introduced IPV in 2016, but vaccination coverage continues to be substandard and limited studies have been conducted in regard to IPV utilization.
- Our study finds that 71% of sampled children aged 12–23 months had received IPV in Kalungu District, which is well below the recommended 95%.
- The household survey found that being encouraged by health workers and Village Health Teams (VHTs) was significant to children’s utilization of IPV; distance to the immunization point and caregiver’s education level were also associated with IPV utilization.
- Qualitative findings from health workers and village health team members further confirmed the perception that distance to the immunization post was important, and village health teams also stated that being encouraged by health workers to immunize was critical to IPV utilization.

In May 2012, the World Health Assembly declared the eradication of poliovirus (WHO, 2012) as a programmatic emergency for global public health. To achieve and sustain a polio-free world, WHO recommended the introduction of at least one dose of inactivated polio vaccine (IPV) given in addition to the oral polio vaccine (OPV). IPV is a systemic immunity booster that eliminates the risk of paralytic polio and circulating vaccine-derived poliovirus-cVDPV, which exist with OPV (Tevi-Benissan et al., 2017). Countries are expected to shift to IPV-only schedules; the double vaccine process is expected to finish by the end of 2020. The introduction of IPV in national programmes has been largely successful despite initial global scepticism; all countries using OPV have now formally committed to adding IPV to their vaccine schedules. Current global polio immunization coverage stands at 85%, against the 90% target (WHO, 2016b), while the overall IPV coverage in sub-Saharan Africa stands at 73% (Anand et al., 2014). As long as a single child remains infected, children in all countries are at risk of contracting polio, and failure to eradicate polio from these last remaining strongholds could result in as many as 200 000 new cases every year within 10 years all over the world (WHO, 2017a).

Uganda is at high risk for polio outbreaks due to the frequent cross-border movements of populations, mainly due to insecurity in neighbouring countries. Wild polio virus may exist in such countries; therefore, the risk of importing and re-establishing these polio cases in Uganda is high (Ministry of Health, 2017). Uganda officially introduced IPV vaccine in May 2016 and integrated it into its routine immunization programme (WHO, 2017b). The current national IPV coverage stands at 60% as of July 2017 (WHO-UNICEF, 2017) while the OPV coverage is recorded at 106%, thanks to efforts to reach recent refugees who were not counted in the initial target population. 

The introduction of IPV in Uganda, like elsewhere, resulted in the delivery of multiple injections during a single visit, with infants receiving IPV alongside pentavalent vaccine (for diphtheria, tetanus and whole-cell pertussis; hepatitis B; Haemophilus influenza type b) and pneumococcal vaccine at 14 weeks (Preza et al., 2017). Infants also receive the third and final OPV dose at 14 weeks. Unanticipated concerns have emerged from other countries over the acceptability of multiple injections, sites of administration and safety. It has also raised common caregiver and provider concerns about the pain experienced by the child, worry about potential side effects, uncertainty about vaccine effectiveness or other misunderstandings about the IPV vaccine (Wallace et al., 2014; Ughasoro et al., 2015).

In Uganda, limited studies have been conducted in regard to IPV uptake since its introduction in May 2016; therefore, understanding the factors associated with IPV uptake among children will help inform policy makers and implementers on better ways to support scaling up of the new vaccine to ensure high coverage and achieve the goal of polio eradication.

## Materials and methods

This was a cross-sectional study, which involved the use of both qualitative and quantitative data collection methods. The aims of this study were to determine the current proportion of children aged 12–23 months utilizing IPV in Kalungu District, and the individual and health service factors associated with IPV uptake.

### Study setting

Kalungu District is one of the new districts that were created in Uganda in 2010, having originally been part of Masaka District. It is a 2 ½ hour drive from Kampala. It has a population of approximately 184 134 people, with 54 360 women of reproductive age and 11 910 children aged 12–23 months, according to the 2014 Uganda National Population and Housing Census (Uganda Bureau of Statistics, 2014). The major economic activities are agriculture, livestock and fish farming; and many people carry out trading on a small scale. The district contains two Health Sub-Districts (HSDs), served by one hospital (offering specialized services), two health centre-level IVs (offering out-patient and in-patient services, maternity, plus an operational theatre), 12 health centre-level IIIs (offering comprehensive out-patient and maternity services) and 15 health centre-level IIs (basic out-patient clinics). All of the hospitals, health centre IVs and health centre IIIs offer vaccination services, as do a few health centre IIs.

The study population was children aged 12–23 months in the selected community in Kalungu District, and respondents were the caregivers of children aged 12–23 months in the community who were 18 years or older, as well as Village Health Teams (VHTs) and health workers at the selected health facilities who had administered vaccinations within the past two years. Caregivers and VHT members who were ill, drunk or inactive (no longer actively carrying out their duties, according to clinic records) were ineligible for participation.

### Sampling

For the quantitative data, sample size determination was done using the Leslie Kish formula since it was a cross-sectional study; it was determined that 410 respondents were required for the study. Multi-stage sampling was used to select the study participants across the two HSDs. A complete list of the sub-counties in each HSD was obtained from the District Planning Unit. A randomizer using computer-generated numbers was used to randomly select two sub-counties from each of the two HSDs. A list of all parishes was obtained from the two selected sub-counties. From these parishes, three were randomly selected from each HSD for a total of six parishes. All of the villages in the selected six parishes were listed, and 20 villages were randomly selected, with 10 selected from each parish representing each HSD.

With the help of the local council leader and VHT, the research team established the number of households with children aged 12–23 months. Then the total number of households in each village was divided by the respective samples to calculate the intervals for household selection. Thereafter, every fifth eligible household was selected. At the household level, the mother or father or other caregiver was selected as a respondent. In total, 21 households were randomly selected from the 20 villages. For the qualitative data, a complete list of the health centres and their levels was obtained from the District Health Office. Then one health centre IV, three health centre IIIs and two health centre IIs were selected because of their high volume capacities in regard to immunization. Interview participants were then selected who were associated with these facilities.

Participants were purposively selected based on their expert opinion and work experience in relation to immunization activities. A total of nine respondents were selected. These included three VHT members who were actively involved in the immunization activities in the selected parishes, four nurses who were directly responsible for immunization activities (two from each selected HSD and sub-county), a District Vaccines Focal Officer and an Assistant District Health Officer responsible for Maternal Child Health (ADHO-MCH).

### Data collection

Home-based records (vaccination cards) were used as evidence for immunization and in case of missing or incomplete home-based vaccination record, recall or verbal history of vaccination by the caregiver was used. The caregiver was asked questions about the site of injection, when the vaccine was received and number of injections administered; answers to two questions were considered sufficient as record of vaccination. A semi-structured questionnaire was used by an interviewer to collect information on the demographics, knowledge and perceptions of the respondents about IPV, and factors related to IPV uptake. A caregiver was considered to have a favourable attitude towards IPV if they agreed with three or more positive statements about it. Interviews of health care providers and VHTs were conducted using a key informant guide. They were audio-recorded and lasted approximately 30 minutes each. Interviews with health workers were conducted in English; interviews with VHT members were conducted in the local language, Luganda, by the trained research assistants, and later translated into English for analysis.

Pre-testing of tools was done in one of the health centres not selected to participate in the study, to check for clarity, content validity and ability to generate required data. After pre-testing, tools were slightly revised before actual data collection.

### Data analysis

Quantitative data were coded and analysed at univariate, bivariate and multivariate levels. Categorical variables were summarized using frequencies and proportions. Continuous variables were summarized using medians with their interquartile ranges (IQR). Chi-square test was used to examine the associations between the selected independent variables and uptake of IPV. Modified Poisson regression with robust variances was used for bivariate and multivariate analysis to identify factors associated with uptake of IPV. Associations were tested at a 95% confidence interval (CI). Prevalence ratios (PR) were used instead of odds ratio because the prevalence of the outcome of interest (uptake of IPV) was >10%, therefore use of an odds ratio would overestimate the strength of association (Wilber and Fu, 2010).

Factors that had a p-value of >0.05 at bivariate analysis were included in the multivariate analysis to obtain adjusted prevalence ratios. Multivariate analysis was conducted to identify the factors associated with uptake of IPV for caregivers with children aged 12–23 months after controlling for background factors. Model-building was done by a stepwise elimination process that involved adding the variables that qualified for the multivariate level one at a time while dropping those which did not have any significance. This was done to obtain the best model with smaller Akaike’s information criterion (AIC) and the log likelihood ratio closer to zero. Crude prevalence ratios, adjusted prevalence ratios and their 95% confidence intervals (CIs) and p-values are reported in tables.

For interview data, thematic content analysis using a deductive approach was used. Each of the transcripts was carefully read to initially become familiar with the content. A sample of the transcripts were chosen and re-read to identify the key points raised so as to enhance data coding. All transcripts were then read again and coding was applied independently by two reviewers, who met to discuss disagreements and come to consensus.

Based on the findings emerging from the data, similar codes were grouped into sub-themes. Similar sub-themes were then merged together and aligned with our initial codes from the conceptual framework. A master sheet containing the themes, subthemes and issues raised was developed and used to obtain the frequently mentioned items. Descriptive statistics were then used to summarize responses from all the interviews in each theme. The most outstanding quotes that were representative of the responses from all interviews are presented.

### Ethical considerations

Ethical approval for this study was obtained from the Makerere University School of Public Health Higher Degrees Research and Ethics Committee (HDREC). Permission was granted from the District Health Officer-Kalungu to do research in the lower health facilities and from the in-charge health officers at each health centre. Written informed consent was sought from participants. Participant confidentiality was ensured using assigned study identity numbers instead of names.

## Results

### Background characteristics of the respondents

A total of 406 eligible respondents completed the survey, the majority (86.9%) of whom were female. Their median age was 27 years (IQR 23–32). More than half (59.3%) of the respondents were aged 20–29 years. 52% of the respondents had only a primary level of education (see Table 1). Of the 406 respondents, 84.6% had child health cards and 71.0% of the study children had received the single dose IPV.

**Table 1:** Background characteristics of the respondents (*n* = 406)

Variable	Frequency (n)	Percentage (%)
**Sex**		
Male	53	13.1
Female	353	86.9
**Age**		
15–19	27	6.65
20–29	241	59.36
30–39	104	25.62
40+	34	8.37
**Religion**		
Catholic	219	53.9
Protestant	64	15.8
Muslim	103	25.4
Others (Pentecostal and Seventh Day Adventists)	20	4.93
**Marital status**		
Married	334	82.27
Not married	72	17.73
**Education**		
None	11	2.7
Primary	209	52.0
Secondary	162	40.3
Tertiary	20	5.0
**Possession of an immunization card**		
Yes	244	84.6
No	44	15.4

**Table 2:** Bivariate analysis of individual factors associated with IPV uptake.

Factor	IPV use			
	Yes n (%)	No n (%)	UPR 95% CI	p-value
**Age**			
15–19	22(81.5)	5(18.5)		1
20–29	185(76.7)	56(23.2)	1.25(0.55–2.86)	0.590
30–39	64(61.5)	40(38.5)	2.08(0.91–4.76)	0.084
40+	18(52.9)	16(47.1)	2.54(1.06–6.06)^*^	0.035
**Sex**			
Male	34(64.2)	19(35.9)	1	
Female	255(72.2)	98(27.8)	0.77(0.52–1.15)	0.208
**Religion**			
Catholic	162(74.0)	57(26.0)	1	
Protestant	41(64.1)	23(35.9)	1.38(0.93–2.05)	0.111
Muslim	71(68.9)	32(31.1)	1.19(0.83–1.72)	0.341
Others^*^	15(75.0)	5(25.0)	0.96(0.43–2.12)	0.921
**Marital status**			
Married	238(71.3)	96(28.7)	1	
Not married	51(70.8)	21(29.2)	1.01(0.68–1.51)	0.943
**Education level**			
No education	3(27.3)		8(72.7)	1
Primary	119(55.9)	94(44.1)	1.45(1.12–1.71)^*^	0.013
Post-primary	167(91.8)	15(8.2)	1.51(1.3–2.10)^***^	<0.001
**Knowledge of IPV**			
Knowledgeable	92(93.9)	6(6.1)	1.82(0.57–5.83)	0.310
Poor knowledge	144(96.6)	5(3.4)	1
**Attitude towards IPV**			
Favourable attitude	7(35.0)	13(65)	1
Negative attitude	282(73.3)	103(26.7)	0.41(0.29–0.59)^***^	<0.001

* P < 0.05;

** P < 0.01;

*** P < 0.001.

UPR = unadjusted prevalence ratio. CI = confidence interval.

### Bivariate analysis of individual factors associated with IPV uptake

In bivariate analysis, caregiver age and education level appeared significantly associated with IPV uptake among children. The prevalence of IPV uptake among children whose caregivers were aged 40 years and above was 2.54 times higher compared with those whose any education whose care givers had not attained any form of education (unadjusted PR 1.51, 95% CI 1.3–2.10). Prevalence of IPV uptake among children whose caregivers had attained post-primary education (secondary and tertiary) was 1.51 times higher than that among children whose caregivers had not attained any form of education (unadjusted PR 1.51,95% CI 1.3–2.10). Prevalence of IPV uptake among children whose caregivers had negative attitudes to IPV was 0.41 times lower compared with that for those whose caregivers had favourable attitudes (unadjusted PR 0.41 95% CI 0.29–059); see Table 3. 

**Table 3:** Bivariate analysis of health service factors associated with IPV uptake

Factor	IPV use		UPR 95% CI	p-value
	Yes n(%)	No n(%)		
**Distance to nearest immunization site**				
<5 km	200(82.0)	44(18.0)	1	
>5 km	65(40.1)	97(59.8)	0.52(0.26–1.02)	0.059
**Transport costs to the immunization site (price ranges in ugandan shillings)**				
No cost	132(62.0)	81(38.0)	1	
1000/= to 4000/=	111(80.4)	27(19.6)	0.51(0.35–0.75)^**^	0.001
5000/= to 10 000/=	17(30.9)	38(69)	0.60(0.27–1.32)	0.202
**Encouraged to take child for IPV**				
Never	5(9.3)	49(90.7)	1	
By health worker	154(90.6)	16(9.4)	2.20(2.0–2.27)^**^	0.01
By VHT	86(86)	14(14)	1.11(1.01–1.31)^**^	0.01
By others (peer, husband, close relative)	73(89)	9(10.9)	0.14(0.01–0.26)^*^	0.02
**Vaccines available at all times at the immunization site**				
Yes	247(84.8)	44(15.1)	1	
No	72(62.6)	43(37.4)	1.54(0.67–3.56)	0.312
**Health workers available at all times at the immunization site**				
Yes	232(81.9)	51(18)	1	
No	93(75.6)	30(24.3)	1.43(0.67–3.05)	0.353
**Informed about the vaccination schedule**				
Yes	220(79.7)	56(20.3)	1	
No	90(69.2)	40(30.7)	0.65(0.26–1.62)	0.353
**Ever turned away from the vaccination site due to long queues**			
Yes	126(77.3)	37(22.6)	1
No	199(81.8)	44(18.1)	2.17(0.96–4.90)	0.061
**Health workers receptive at the vaccination site**			
Yes	224(87.5)	32(12.5)	1
No	120(80)	30(20)	0.95(0.37–2.45)	0.920

* P < 0.05;

** P < 0.01;

*** P < 0.001.

UPR = unadjusted prevalence ratio. CI = confidence interval.

### Bivariate analysis of health service factors associated with IPV uptake

In bivariate analysis, incurring transport costs to reach the immunization site and being encouraged by health workers or VHTs to take the child for IPV were significantly associated with IPV uptake (Table 3).

### Multivariate analysis of factors associated with IPV uptake

After controlling for the education level of caregivers and encouragement by health workers and VHTs to take children for IPV, the prevalence of IPV uptake among children whose caregivers resided >5 km away from an immunization post was 0.32 times lower than those who resided <5 km from the immunization post (Adjusted PR 0.32,95% CI; 0.16–0.62) (Table 4).

**Table 4:** Multivariate analysis of factors associated with IPV uptake among children aged 12–23 months

Factor	IPV use		APR 95% CI	p-value
	Yes n(%)	No n(%)		
**Caregivers’ age**				
15–19	22(81.5)	5(18.5)	1	
20–29	185(76.7)	56(23.2)	1.05(0.47–2.31)	0.908
30–39	64(61.5)	40(38.5)	1.04(0.42–2.61)	0.930
40+	18(52.9)	16(47.1)	1.07(0.35–3.24)	0.905
**Caregivers’ education level**				
No education	3(27.3)	8(72.7)	1	
Primary	119(55.9)	94(44.1)	0.59(0.22–1.63)	0.311
Post-primary	167(91.8)	15(8.2)	1.16(1.05–2.22)**	<0.001
**Caregivers’ attitude towards IPV**				
Favourable attitude	7(35.0)	13(65)	1	
Negative attitude	282(73.3)	103(26.7)	0.45(0.26–0.78) *	0.005
**Distance to the nearest immunization site**				
<5 km	266(85.8)	44(14.2)	1	
>5 km	45(46.8)	51(53.1)	0.32(0.16–0.62)**	0.001
**Transport cost to immunization site (price ranges in ugandan shillings)**				
No cost	165(73.4)	51(23.6)	1	
1000/= to 4000/=	141(96.5)	5(3.4)	0.34(0.29–4.1)*	0.009
5000/= to 10 000/=	17(38.6)	27(61.4)	1.55(0.91–2.65)	0.109
**Encouraged to take child for IPV**				
Never	11(18.3)	49(81.6)	1	
By health worker	110(87.3)	16(12.6)	1.24(1.22–3.47)^***^	<0.001
By VHT	126(90)	14(10)	1.1(1.02–1.58)^**^	0.01
By others (peer, husband, close relative)	71(88.7)	9(11.3)	0.45(0.23–1.89)	0.61

* P < 0.05;

** P < 0.01;

*** P < 0.001.

APR = adjusted prevalence ratio. CI = confidence interval.

After controlling for distance to the immunization post and encouragement by health workers or VHTs to take children for IPV, the prevalence of IPV uptake among children whose caregivers had attained post-primary (secondary and tertiary) education was 1.16 times higher than among children whose caregivers had not attained any education (Adjusted PR 1.16,95% CI; 1.05–2.22). Children whose caregivers were encouraged by health workers or VHTs to take children for IPV were 1.24 times more likely to utilize IPV vaccines than those whose caregivers were not encouraged to do so.

### Qualitative findings

Findings from the surveys were supported by qualitative interviews. Data were organized into key themes: distance to immunization post; and encouragement to utilize IPV services.

Respondents reported that children whose caregivers resided far from the immunization post were not effectively utilizing IPV immunization services, as revealed in the quotes below:

*… our health facilities are not evenly distributed; caregivers in hard to reach areas are really struggling since they have to incur high transport costs. This deters the children from utilizing these services, but we ensure that outreaches are conducted routinely amidst the financial and human resources challenges* (ADHO-MCH).

*… The mothers who stay far find it a challenge to take their children for immunization* (VHT, HC III).

*… caregivers staying in hard to reach areas such as landing sites [collection and trading centres for fish] sometimes fail to make it to the immunization point due to constraints in transport as they try to cross the water* (VHT, HC III).

Respondents also stated that children whose caregivers had been encouraged by the health workers and VHTs were utilizing the vaccine more than those who had not been, as illustrated in the quotes below:

*… we start our health education when the mothers come for antenatal care. The VHTs help us to follow them up and we encourage those mothers in our neighbourhood. This helps us to ensure that the children utilize the IPV immunization services* (Nurse, HC II).

*… some caregivers still have misconceptions about the polio vaccine of course, some wonder why the change from oral to injectable; but we continuously health educate and encourage them to have their children immunized for their good. Surely most of them adhere since they trust us and some have even witnessed the consequences of not immunizing against polio* (District focal person for Expanded Programme on Immunization).

## Discussion

### Proportion of caregivers to children aged 12–23 months utilizing IPV

This study found out that the current proportion of IPV uptake in Kalungu District stands at 71%. This is slightly higher than the last reported national and district coverage figures in 2017, which were at 60% and 54.4%, respectively (KDLG, 2017; WHO and UNICEF, 2017). However, the proportion of IPV uptake observed in this study was slightly lower than the overall IPV coverage in sub-Saharan Africa, which is at 73% (Anand et al., 2014), and is lower than many other regional coverage figures such as 80% for Rwanda (UNICEF, 2018b) and 90% for Kenya (UNICEF, 2018a). In an earlier study in Kenya, the high prevalence of IPV uptake was attributed to the mobile tracking system, whereby mothers are sent SMS/text reminders for their next immunization date, coupled with the traditional home visits, which are believed to reduce dropout rates. However, the increasing IPV prevalence in Kalungu from 54.4% in 2017 to the current 71% indicates a positive trend and could be attributed to the increasing awareness about the vaccine among the caregivers, coupled with the routine and supplementary immunization activities conducted at both health centres and outreaches.

### Individual factors associated with IPV uptake

This study found that the education levels of caregivers were significantly associated with IPV uptake. This is consistent with a study conducted in Nigeria where caregivers with lower education levels were linked to low vaccine uptake among children. Similar findings from a study conducted in Kenya and Ethiopia (Sullivan et al., 2010) on the factors associated with immunization completion rates indicated that children whose caregivers had attained some level of education had higher chances of following and completing the immunization schedules compared with those whose caregivers had not attained any level of education (Abuya et al., 2011). This could be attributed to the fact that caregivers who have attained some form of education can more easily comprehend health information and may be more aware of the benefits of child immunization.

This suggests that education has a strong influence on the uptake of public health interventions in general, since many public health topics are taught in schools, and may empower future caregivers to protect their children from preventable diseases. Therefore, there is a need to reinforce sharing of health information about immunization in schools so as to widen the knowledge base of future parents.

Findings from this study show that caregiver religion was not significantly associated with IPV uptake among children, which contrasts earlier studies conducted in other African countries, like Nigeria, where religious beliefs in relation to polio immunization negatively affected vaccine uptake (Obadare, 2005). These studies found that many in the Muslim community in Nigeria believed that polio campaigns were a Western conspiracy to control the Muslim population and that polio vaccination was used as a tool to cause sterility in children. Some studies have also suggested that poor uptake of immunization services in the Muslim community may have cultural underpinnings in addition to the mistrust of vaccinations (Ophori et al., 2014). However, while the finding was different in this study, there may still be a benefit in the active involvement of the different religious leaders to increase their awareness about the vaccine and advocate for its uptake among children.

### Health service factors associated with IPV uptake

Distance to the immunization site, along with incurring transport costs, was found to be significantly associated with IPV uptake among children aged 12–23 months. This is consistent with findings from a study conducted in Bangladesh where caregivers close to health centres had higher uptake of IPV immunization services compared with those who resided far away (Breiman et al., 2004). Further, comparable findings from studies conducted in Uganda and India revealed that rural areas, which have fewer immunization posts, had lower vaccination coverage than urban areas (Babirye et al., 2012; Bbaale, 2013; Obregón et al., 2009). Having to travel long distances may be challenging for many caregivers if it impacts on their productive time needed for domestic or commercial duties. Caregivers may be discouraged by the long distances or by the transport costs they have to incur in order to access the health centres. There is a need to reduce transport costs, as well as to improve outreaches to serve the children who are far from the health facilities and bring services closer to them for easier uptake.

In this study, being encouraged by health workers or VHTs to take children for IPV vaccination was found to be significant. These findings are consistent with earlier studies conducted in Nigeria (Tabana et al., 2016; Osadebe et al., 2017) where children utilized immunization services more after their caregivers were encouraged by health workers. In a study in Bangladesh, caregivers (about 48%) attributed their acceptance of IPV to the fact that health care practitioners had encouraged them to immunize their children, stating that the health workers knew best in regard to the wellbeing of their children (Estivariz et al., 2017). This is also similar to findings from a post-IPV introduction study conducted in Albania, where caregiver acceptance of IPV was attributed to trust in the immunization system due to health workers’ recommendations; therefore, having a strong trust in health workers or other community health workers is an important enabling factor towards uptake of IPV among children (Platt, 2015).

The study findings are also consistent with several other studies conducted in Tanzania, Bangladesh and other developing countries on the influence of families and peers on immunization uptake: namely, having friends and family members with positive immunization views resulted in improved immunization uptake, underscoring the role of social support in improving immunization outcomes (Keoprasith et al., 2012; Stockwell et al., 2014; Brunson, 2015; Mazige et al., 2016). When caregivers are confident that health worker or VHT recommendations are for the good of their children, they may have less hesitancy to follow such recommendations. Therefore, health caregivers and VHTs have a vital role in promoting such public health interventions, not only to caregivers of young children but to the entire community.

There is therefore a need to intensify the health education at health facilities and in the community along with strengthening the traditional home visit system so as to continue encouraging caregivers to take their children for IPV immunization.

## Conclusions

The current proportion of IPV uptake among children aged 12–23 months in Kalungu District, Uganda is higher than the last reported national coverage, but more progress needs to be made. Caregivers’ education status was significantly associated with IPV uptake, as was distance to the immunization post. Health workers and VHTs in the community have a critical role in promoting uptake of the vaccine. Strengthening multiple aspects of the health system together can have a beneficial impact on IPV uptake and improve the health of Uganda’s children.

*Conflict of interest statement*. None declared.

*Ethical approval.* Ethical approval for this study was obtained from the Makerere University School of Public Health Higher Degrees Research and Ethics Committee (HDREC). Permission was granted from the District Health Officer-Kalungu to do research in the lower health facilities and from the in-charge health officers at each health centre. Written informed consent was sought from participants. Participant confidentiality was ensured using assigned study identity numbers instead of names.
